# Bluetooth Low Power Modes Applied to the Data Transportation Network in Home Automation Systems

**DOI:** 10.3390/s17050997

**Published:** 2017-04-30

**Authors:** Josu Etxaniz, Gerardo Aranguren

**Affiliations:** Electronic Technology Department, University of the Basque Country, Bilbao 48980, Spain; gerardo.aranguren@ehu.es

**Keywords:** ACL, Bluetooth, Delay, Empirical model, Home Automation, Latency, Multi-hop, Scatternet

## Abstract

Even though home automation is a well-known research and development area, recent technological improvements in different areas such as context recognition, sensing, wireless communications or embedded systems have boosted wireless smart homes. This paper focuses on some of those areas related to home automation. The paper draws attention to wireless communications issues on embedded systems. Specifically, the paper discusses the multi-hop networking together with Bluetooth technology and latency, as a quality of service (QoS) metric. Bluetooth is a worldwide standard that provides low power multi-hop networking. It is a radio license free technology and establishes point-to-point and point-to-multipoint links, known as piconets, or multi-hop networks, known as scatternets. This way, many Bluetooth nodes can be interconnected to deploy ambient intelligent networks. This paper introduces the research on multi-hop latency done with park and sniff low power modes of Bluetooth over the test platform developed. Besides, an empirical model is obtained to calculate the latency of Bluetooth multi-hop communications over asynchronous links when links in scatternets are always in sniff or the park mode. Smart home devices and networks designers would take advantage of the models and the estimation of the delay they provide in communications along Bluetooth multi-hop networks.

## 1. Introduction

Home automation [1] is an emerging vision that offers efficient home management system with convenience, comfort, energy efficiency and security. Generally speaking, it is defined as the introduction of technology inside the home to enhance the quality of life of its occupants, by providing them with different services such as smart health [2], tele-health [3], multimedia entertainment or energy conservation [4]. 

Home automation solutions can be conservative or wireless. The conservative home automation solutions are usually based on power line or wired communication technologies. They are complex, expensive, inflexible, and involve time-consuming installations. On the other hand, wireless home automation architectures have gained popularity in home automation for numerous advantages such as plug and play nature, flexibility, interoperability and cost effectiveness. 

Usually, wireless approaches comprise smart devices, mainly wireless embedded sensors and actuators, which communicate with each other directly or via a centralized server to achieve defined automation functionalities [5], usually monitoring and control applications for home user comfort and efficient home management [6]. Devices with sensing and recognition capabilities as well as collection and processing are typically a part of home automation systems. Such systems are a multidisciplinary approach [7], as they require the convergence of many areas of Computer Science in order to fulfill the purpose of improving the quality of life of the residents. 

Then, electronic systems to interconnect nodes and communicate the measured data can be found in many home automation deployments [8]. Here, every device must be capable to transmit and receive the context information that is collected by the sensors. However, since in many homes the context information usually changes at low pace, the continuous sensing of the information is not mandatory. Thus, the nodes can sleep at regular intervals. In fact, home automation deployments composed of wireless multi-hop networks frequently require long-life sensor nodes to be interconnected through a communication network. Therefore, working with devices in low power mode is almost mandatory in home automation deployments [9]. 

The nodes can be connected to the electricity supply so that there is no need to worry about the power consumption of the nodes. In addition, if a battery is added to the node, the node will be fully operational in the case of blackouts. In the case there is no chance to connect the nodes to the electricity supply, the nodes must include a battery power supply. Moreover, they must work in low power mode so that the lifecycle of the node is as long as possible. The inactivity intervals of the gateway and sensor nodes that work in low power mode can be scheduled to reduce their power consumption and, hence, the consumption of the whole network. However, the inactivity intervals degrade significantly the time performance of the network. Thus, whether the nodes included in the network are connected to the electricity supply or not, it is important to reduce the power bill of the home automation deployment without degrading significantly its performance in terms of response time or latency. 

Actually, the analysis of the time performance of wireless networks is a key point in the design and management of such networks [10]. It is a fact that the transmission medium in wireless communications is usually unreliable. However, the use of wireless networks for limited response time applications is increasing. As a result, latency is a key metric when defining the Quality of Service (QoS) of a communication system [11]. 

Any application that shares the measured data for their collaborative use is considered here. There is a wide range of applications that satisfy this feature. However, this paper focuses on home automation deployments [12]. 

There are many wireless communication standards to implement home automation networks [5]. Bluetooth is a worldwide communication standard without radio emission license cost. It allows for multi-hop networking with low energy consumption. Such features make Bluetooth the chosen wireless technology as the communication standard to implement multi-hop home automation networks. Even though there are differences among the releases of the Bluetooth standard, the chosen release, 2.0, is still on the cutting edge of the technology in terms of multi-hop transmission performance [13]. 

Then, focusing on obtaining the data that lead to the empirical model for the latency in the wireless communications, a test bench based on commercially available off-the-shelf (COTS) products was developed. Several nodes were developed with some Bluetooth communication modules. Such nodes are integrated into electronic systems for home automation deployments so that they can interconnect via wireless links. Moreover, the middleware to manage the performance of the node at the Host Controller Interface (HCI) level of the radio module was built, and the nodes were configured using high-level commands. Subsequently, the performance of Bluetooth gateways included in multi-hop networks was analyzed in the tests by measuring the end-to-end latency QoS metric. The data were analyzed statistically to limit the errors made in examining a finite set of samples, and the tests were repeated a lot of times. 

As a result, this paper analyzes the end-to-end latency of the multi-hop communication network to estimate the time performance of the transportation network for home automation systems. Several Bluetooth low power modes for the radio module of the nodes are available. Two typical Bluetooth low power modes have been considered here: sniff and park. The sniff mode keeps the biggest activity when nodes are in low power mode; and the park mode loses the most connectivity while nodes are parked. 

To the best of the authors’ knowledge, there is no empirical latency analysis in Bluetooth low powered multi-hop communications. Therefore, this paper introduces the empirical models for the transmission latency from end-to-end in Bluetooth multi-hop wireless networks when working with Asynchronous Connectionless Links (ACL) in both, park and sniff low power modes. The models will benefit the home automation systems and wireless networks designers providing them with the estimation of the latency that every hop in the communication adds. 

The rest of the paper is organized as follows. Section 2 introduces a case study of wireless home automation networking. Furthermore, the multi-hop networking capability of Bluetooth standard and the way it is applied to such systems are explained. Next, Section 3 gives the features of the test bench developed in this research. In addition, Section 4 explains the details of the methodology of the tests. In Section 5, the results are discussed. After that, Section 6 proposes some models for the latency in the case study of multi-hop wireless communications networking. Finally, the conclusions are summarized and the future work pointed out in Section 7.

## 2. Home Automation Systems 

The latest technological advances in different areas such as embedded systems, sensing, ubiquitous communication technologies, or wireless communications are driving automation systems inside smart homes [7]. Such systems should be anticipatory, adaptive, and embedded as well as transparent, responsive, and sensitive. 

Next, a case study to show the wireless networking for home automation deployments is introduced. After that, the technical details of Bluetooth standard to allow multi-hop networking are given. Then, the state-of-the-art literature about Bluetooth networking in home automation systems is reviewed. 

### 2.1. Case Study: Networking for Home Automation Deployments

**Figure 1:**
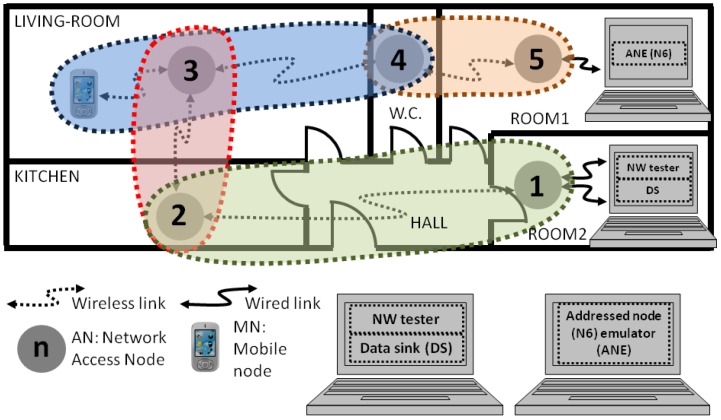
Example of multi-hop network deployment for the data transportation network in home automation solutions.

The practical scenario of the multi-hop network deployment for a home automation solution represented in Figure 1 is considered in this paper. Such network could also be deployed in assisted living homes, hotels, hostels, student residences, foster homes, or any other analogous buildings. 

The network includes some wireless access nodes (AN_i_) and mobile nodes (MN_ij_), as shown in Figure 1. The MN_ij_, as the AN_i_, would include some sensors and actuators depending on the application of the network. The AN_i_ in Figure 1 are deployed all through the home to provide wireless network connectivity to the MN_ij_. Many types of sensor are included in home automation solutions (security system or alarm, smoke detector, CO_2_ level detector, glass break sensor, window open sensor, motion or presence detector, door open sensor, smart door lock, air conditioner, humidity sensor, temperature sensor, luminance sensor, occupancy sensor, light controller, etc.). They can be attached to both access and mobile nodes with wireless connectivity. 

The nodes would centralize the measured data into the data sink (DS). The processing of the data gathered while monitoring would generate some alarms and would require some control commands. Both commands would be generated in the DS. Furthermore, several functionalities in the computer connected to the first and the last nodes of the network would ease the monitoring process. For instance, the test commands would be generated in the network tester (NW tester) and the resultant data would be aggregated in the DS. 

Many reasons can be found to deploy a wireless network of access nodes instead of considering the wired solution. On the one hand, the deployment costs of a wireless network are lower than the costs of the wired approach. On the other hand, the installation of the network access nodes is complex, and might be risky. Moreover, the wired solution is usually inflexible. Thus, a wireless network is arranged whenever the budget restrictions, flexibility, convenience, or other issues make this approach the most suitable one. 

Generally speaking, the devices in home automation solutions are interconnected and they form a communication network. Therefore, a communication standard must be found to provide connectivity to such devices, and Bluetooth [14] is a standard that satisfies these requirements. 

Then, in the case shown in Figure 1, every MN_ij_ connects to the most appropriate AN_i_ periodically, once the programmed inactivity period of the node is finished. The requirements in latency depend on the specific application and the type of sensor. As an example, temperature or humidity sensors are usually included in applications with longer monitoring intervals than presence sensors. If the wireless communication standard offers many low power working modes, as it happens in Bluetooth, it is necessary to choose sensibly the low power mode for the best performance of the system. 

### 2.2. Bluetooth Multi-Hop Networking 

The Bluetooth networking capability is based on piconets, i.e., the smallest network that can be formed with this communication technology. Whenever two or more Bluetooth devices share a radio channel, they form such centralized networks called piconets. A central node (master), which controls the communications, and up to seven active nodes (slaves) are included in piconets. Figure 1 shows some piconets. For instance, the one colored in dark blue, where, for i = 3, the AN_i_ is the master, and the AN_i + 1_ and the MN_i1_ are the slaves (only the MN_3_ is depicted).

The standard takes in the notion of a more complex network, i.e., the scatternet, but it does not define the way to form it. Such network not only helps to extend the coverage of wireless devices, but also increases the amount of active nodes in a limited area. Any Bluetooth device can play the role of slave in many piconets as well as be the master in at most one piconet. When some nodes operate in two or more piconets, they are called gateways and a multi-hop network is formed, i.e., a scatternet. 

Bluetooth defines two types of gateways according to the roles they play in the piconets: master-slave and slave-slave. The master-slave gateway offers less delay from end to end for traffic between two adjacent piconets (inter-piconet traffic) [15]. Since the research is focused on inter-piconet traffic delivery through scatternets, the nodes in the test bench were configured as master-slave gateways, i.e., as masters of the piconet formed with the next node, and as slaves with the previous node (according to the numbers naming the nodes in Figure 1). 

The gateways in Figure 1 are connected in linear topology and they relay the data gathered from both the sensors connected to the AN_i_ in the rooms and the sensors connected to the MN_ij_, which are carried by the monitored users. The first sensors can be those to measure the temperature, detect the presence of people, or even presence of smoke; and the second ones to measure the vital signs or user’s body temperature. 

Then, every intermediate node in the wireless test bench, i.e., every gateway, participates in two piconets given that it has an open link as a master and another link as a slave. The communications in Bluetooth follow time division multiplex (TDM) scheme defined by the master node. At the baseband level, each slot lasts 625 μs, so the slot rate is 1.6 kHz. Thus, there are inactivity intervals for the nodes inside the piconet. When two nodes establish a link, they negotiate the moments of activity in the piconet and the intervals in which each slave will be transmitting and receiving data. The master node of every piconet in the tests polls slave nodes every 25 ms, as it is set by default. Thus, nodes can take advantage of the activity breaks in the piconet, where they are slaves, to attend to the piconet where they play the master role, guaranteeing that they can attend to both of the links. Not only the slave nodes but also the master nodes can abandon the piconet while there is no activity and start playing the slave role in the other piconet. However, if a node perceives the absence of the other node in the piconet, the node closes the link and the link must be re-established later if it needs to transfer data to the other node. Hence, the latency of the multi-hop communication will increase in such situations. 

The standard defines the Basic Data Rate that allows up to 723.2 kbps asymmetric rate with 1 bit per symbol transmission under Gaussian Frequency Shift Keying (GFSK) modulation. In addition, the standard defines the sniff and park low power modes to take advantage of the inactivity intervals. Both modes are analyzed in this paper. 

On the one hand, when any node operates in the sniff mode, the inactivity intervals (T_sniff_) or periods of absence in the piconet are configured such that the slave node agrees with the master to periodically listen to its transmissions. On the other hand, when the nodes operate in the park mode, some beacons are listened by the slave node every inactivity interval (T_park_). Meanwhile, the node can be low powered or dedicated to other tasks out of the radio tasks in that piconet. 

### 2.3. Bluetooth Applied to Home Automation Systems

The requirements for a wireless home automation system are fulfilled by Bluetooth standard [14]. This technology has been included in the data transportation network of many home automation solutions [16,17,18,19,20,21]. In fact, Bluetooth protocols have significant potential to support deterministic behavior, i.e., real-time, asynchronous communication [22]. 

Many issues give relevance to the analysis of the wireless networks performance. First, the use of wireless networks for constrained response time applications is increasing. Next, the transmission medium is usually considered as inaccurate. Finally, the latency increases randomly due to both, the retransmissions that the medium inaccuracy leads to, and the unpredictable delay between the delivery of data to the host interface and radio transmission. Therefore, the latency shows a non-trivial variability [10] that should be analyzed. 

Because of the efforts on Bluetooth, an extensive literature is available on many aspects of Bluetooth networking. Next, the state-of-the-art of Bluetooth networking performance analysis is summarized. The performance is evaluated with QoS metrics. There are two types of metrics: traffic dependent or traffic independent [23]. The first ones involve the specification of a defining source, packets destination and traffic profile. In addition, packet flows are considered. Consequently, the evaluation of traffic dependent metrics is difficult, and traffic independent performance measures are frequently used.

The most common of traffic independent performance metrics are the bit error rate (BER), the throughput and the latency. For example, the first two of these metrics, i.e., the BER and throughput, were analyzed under noise and interference conditions [24] in scatternets. Furthermore, a mathematical model to analyze the performance of Bluetooth data links was provided [25], in terms of many metrics, latency included. Some other analytical approaches to the performance of piconets were validated with simulation engines [26,27,28]. However, none of the results in these researches was validated in hardware test benches so that the model proposals get close to the real world performance. 

In addition, some theoretical models to define the communications inside piconets were already proposed. For example, the latency in asynchronous communications in piconets was studied [29], as well as analyzed when particularized for connections using serial port profile [30]. Moreover, the latency in the communications in a piconet follows a step outline with the length of the data packets as a result of the data segmentation [31]. All of them were empirically validated in actual piconets. 

On the other hand, the file transfer delay (FTD) was introduced in a piconet. It included the time delay of the packet, and the delays due to encapsulation/de-encapsulation, signal propagation and retransmission. Then, an empirical model was proposed to foresee the FTD in a piconet without theoretical support [32].

Generally speaking, the latency in scatternets has barely been analyzed. However, the latency in a three node scatternet was determined [33]. It included a slave–slave gateway. The authors pointed out that the communication between a master and a slave node is not symmetric and involves different latency values. However, a real world scenario should include more than one hop. 

Real world home automation solutions designers need models of the main metrics of wireless networks, so that they can determine the application range of a specific wireless standard. Actually, low power modes with a defined period of inactivity are almost mandatory in wireless home automation deployments. Theoretical calculations and simulation engines cannot foresee all the real-world issues; thus, empirical models are required to analyze the viability of specific wireless applications [34]. In fact, a few empirical models on latency have been obtained in Bluetooth multi-hop networks [13,35]. Then, since Bluetooth does not support synchronous links in scatternets, empirical models to define the latency in scatternets with ACL links in low power modes are necessary. 

## 3. Test Bench

The goal of this research is to study the latency on multi-hop communications, including as many gateways as possible in a scatternet. Then, the first step towards the goal consisted of the implementation of the proprietary nodes with Bluetooth connectivity. Next, the wireless test bench was deployed so that the test bench consisted of a multi-hop wireless network of proprietary nodes based on Bluetooth technology.

**Figure 2:**
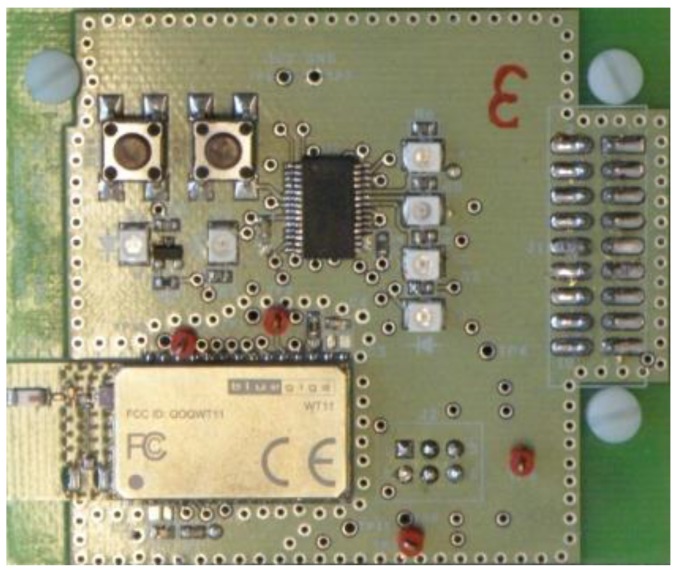
Photograph of one of the implemented Bluetooth nodes.

One of the implemented proprietary nodes can be seen in Figure 2. The nodes included the WT-11 chipset of Bluegiga [36], general-purpose input and output components, and some test pins. The chipset included a firmware developed by Cambridge Silicon Radio [37], named BlueCore4 [38], that eases the low power modes defined in the Bluetooth specifications and analyzed in this manuscript, i.e., park and sniff. In addition to the firmware, a proprietary middleware was designed using BlueLab [39], the software tool to develop embedded software given by the manufacturer of the chipset [37]. BlueLab eases the programming of the nodes at the HCI level, the lowest level that any proprietary middleware can access within the Bluetooth protocol stack. The middleware included the processing tasks, which were limited to the most basic operations to restrict their influence in the latency. Moreover, the middleware integrated processes to initialize the communications, present the operation status and route data to other nodes. 

**Figure 3:**
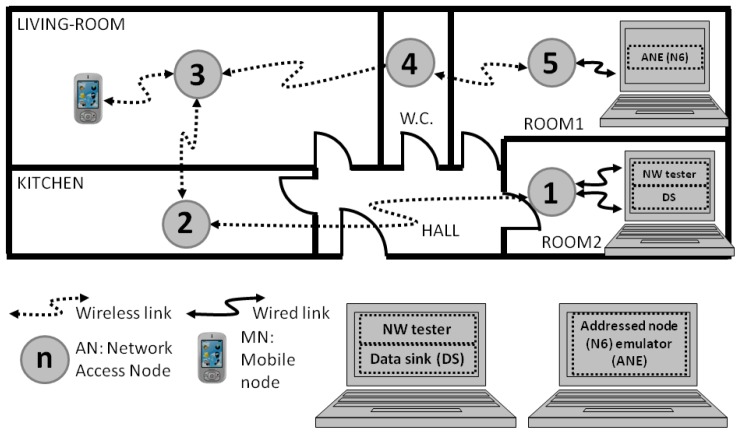
Example of multi-hop network deployment for a home automation solution.

This research considers the deployments of multi-hop networks based on Bluetooth communication standard inside homes and buildings. An example of such deployments can be seen in Figure 3. The grey circles represent the network access nodes deployed. Some sensors (smoke, temperature, etc.) would be attached to the nodes, and the network could be accessed by wireless devices, such as mobile phones. Even though the electricity supply is usually at hand in such indoor deployments, a battery is included in each node to overcome any failure in the electricity supply that might happen.

Every pair of nodes in the test bench built a piconet. In addition, the interconnection of piconets built a multi-hop network with linear topology, which is one of the simplest topologies available. However, the linear topology is the most suitable one to study the performance of the desired amount of Bluetooth gateways with the least amount of nodes. 

Here, every intermediate node operates as gateway and relays the data gathered from other sensor nodes (not depicted in Figure 3) to the DS. The DS stores all the information available in the system and shares it with all the devices through the multi-hop network. 

On the other hand, there is a network administrator who accesses the data in two ways. They can gain admission to the network through a mobile phone with Bluetooth connectivity or through a computer connected to the node labeled as number one. 

## 4. Methodology

The tests were performed in the test bench deployed in the laboratory of the research group, and the nodes were spread in the laboratory and the corridor nearby. First, a key point was checked, i.e., the medium range distances considered in the next tests would have no significant influence in the latency of the communication. Then, the methodology to analyze the performance of master-slave gateways in scatternets described in [40] was applied. 

Furthermore, the proprietary middleware in the nodes of the test bed included not only the specific values to define some of the parameters for the low power modes under test, but also the one to operate with the Basic Data Rate. Moreover, the way to interconnect the nodes during the tests for the case of four hops in the communication is shown in Figure 3. A computer was connected to the end nodes, i.e., the first and last ones of the network. The DS, the NW tester and the addressed node emulator (ANE) functionalities were built-in the same computer and, hence, they were synchronized. As a result, not only the time that the data packets took hopping from master to slave nodes could be measured, but also the time taken from slave to master nodes. 

Then, the NW tester functionality sends ping type data packets to the last node of the wireless test network, i.e., node N6 in Figure 3, and the intermediate nodes receive the packets, process and resend them towards the addressed node. The ANE functionality in the computer receives the ping packet, logs the reception moment and sends the answer packet while logging the transmission moment. The answer consists of a pingback type data packet that flows along the network, going from slave to master nodes of the piconets and, finally, it reaches the computer connected to the first node of the network. The DS functionality included in the computer logs the reception moment of the pingback packet. 

Therefore, each ping packet went hopping from node to node until it reached the destination node, and the pingback packets went back to the source node following the same path. The next ping packets were sent right after the pingback packet reached the source node. This way, the ping packets were sent randomly because the sending interval was defined by the latency of the network, which turned to show some variability in the tests. 

The latencies for both the ping packets (master-to-slave communication) and the pingback packets (slave-to-master) are obtained through the calculation of the differences between the logs in both ends of the network. Moreover, since the communication follows the TDM scheme, both the ping and pingback packets fit in a time slot defined in the Bluetooth standard (625 µs) [14]. The tests were repeated numerous times (in total, 12,775 samples were taken in the park mode and 10,828 samples in the sniff mode). 

**Figure 4:**
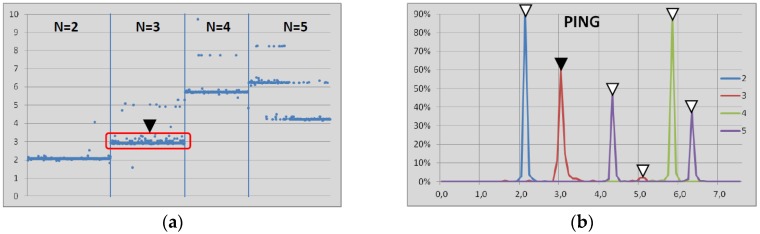
Example of the analysis of the set of latency samples measured in the tests. (**a**) Set of samples for the case of sending ping data-packets through from 2 to 5 hops (N) while operating in the sniff mode with 2 s of inactivity interval. The red square gathers the samples inside a subseries identified as the latency when the ping data-packet takes three hops. (**b**) Histograms of the samples for different amount of hops for the same cases, where the subseries of samples are identified with the white triangles.

Then, once the whole set of samples is acquired, it was analyzed following the next steps. First, the set of latency samples was depicted, as shown in Figure 4a for the case of sending ping data-packets through from 2 to 5 hops while operating in the sniff mode with 2 s of inactivity interval. There, some subseries of samples can be identified, as shown with the red square in Figure 4a. They correspond to the latency in the communication through some specific amount of hops. Next, the histograms of the samples for different amount of hops were depicted, as shown in Figure 4b, where the subseries of samples are identified with higher definition. For instance, the black triangle points out the same subseries in both representations (Figure 4a,b). Other subseries are identified in Figure 4b with white triangles. Then, the average value of the subseries is extracted for each one of the tests and such values are summarized into a data table, which are detailed in Section 5. The data table includes the low power mode, the amount of hops, the inactivity interval and the latency measured for both the ping and pingback data-packets. The academic background and the state-of-the-art literature are the basis for the models proposed to define the latency values in the data table. Four models are obtained in this paper through linear multiple regression [41]: in the sniff and the park mode, for both ping and pingback data-packets. 

Since the set of samples showed that the retransmissions might happen, they should be taken into account and, therefore, the models included the probability of retransmissions during the multi-hop communication. The retransmissions are usually a result of the influence of a series of external factors, as the other processes run by the operating system in the computer, or the presence of other equipment in the same frequency band that interfere the wireless communication. However, as the actual values of the probability must be estimated in the actual home automation application, they are not included in the four numerical models given in this paper. Finally, the models are validated with R^2^ statistical factor [42] and the mean squared error (MSE) [43]. 

Overall, the addition of the estimation of the probability of retransmission for the actual home automation application to the specific model to be considered will provide a more precise definition of the latency that the system will show while operating. This latency definition helps the network engineers to design the management of limited response time systems, and, more precisely, wireless home automation systems, when operating in sniff and park low power mode. 

## 5. Discussion of the Results

This paper analyzes the latency in multi-hop networks with Bluetooth standard, so the time that the ping type data packet takes from source to destination node, as well as the time that the pingback data packet does, were measured. The amount of intermediate nodes in the network, and hence the amount of hops, was changed. In addition, several periods of inactivity (from 0.1 to 2 s) of two Bluetooth low power link policies were considered (park and sniff). An average of 320 samples per situation under test was considered in the park mode, and 270 samples per situation in sniff. 

Several data tables with the results of the tests are available to analyze the latency in Bluetooth multi-hop networks. Table 1 summarizes the results of the latency tests vs. amount of hops for the master-to-slave communication (δ_PING_) when links in the network are always in the park mode, as well as the results when the communication is from slave to master (δ_PINGBACK_). Four inactivity intervals (T_PARK_) are considered, namely 0.1, 0.5, 1 and 2 s. The table includes the values properly identified from the results of the tests. A monotonically increase of the latency when the amount of hops is increased can be seen in Table 1. Moreover, as expected, the results of latency when the nodes are operating in the park mode show higher values in the pingback case (i.e., data packets going from slave to master) when compared to the ping case (i.e., data packets going from master to slave). In addition, the latency from end-to-end in Bluetooth multi-hop wireless communications when links are always in the park mode exhibits linear dependence with the inactivity period of the nodes.

On the other hand, Table 2 summarizes the results of the latency tests vs. amount of hops for the master-to-slave communication when links in the network are always in the sniff mode, as well as the results when the communication is from slave to master. As in the park mode, four cases of inactivity intervals were considered, namely 0.1, 0.5, 1 and 2 s. 

Table 2 shows that the latency with ping data-packets when links are in the sniff mode is very close to the latency with pingback data-packets, i.e., there is no major difference between these two latencies. The reason is that the sniff mode can be exited by either the master or the slave sending a specific request command so that the requested device replies with the according acceptance command. In addition, not only a monotonically increase of the latency when the amount of hops is increased can be seen in Table 2, but also the latency from end-to-end in Bluetooth multi-hop wireless communications when links are always in the park mode exhibits linear dependence with the inactivity period of the nodes.

## 6. Models for the Latency

The results of the tests are the basis of the models for the latency in asynchronous communications along Bluetooth multi-hop networks when master–slave gateway nodes are in sniff and park low power modes. The models describe and calculate the latency in Bluetooth networks with linear topology.

The procedure to obtain the models consisted of analyzing the results and extracting the dependences of the latency with the parameters tuned in the tests, i.e., the amount of hops of the communication and the period of inactivity. Then, the coefficients of the master equation were defined for the cases under study, and linear multiple regression was performed to calculate them. Afterwards, the models were defined and validated with the measurements through the R^2^ statistical factor and the MSE. On the one hand, R^2^ is used in the context of a statistical model which pretends to predict future results or to test a hypothesis. It determines the quality of the model to replicate the results and the part of the variation of the results that can be explained with the model [42]. On the other hand, the MSE is a measure of the quality of an estimator that measures the average of the squares of the errors or deviations, i.e., the difference between the estimator and what is estimated [43]. 

### 6.1. Bluetooth Park Low Power Mode

Taking Table 1 as reference, the latency from end-to-end in Bluetooth multi-hop networks when links are always in park low power mode can be modeled as in Equation (1), where some of the time contributions described in [40] are included, namely the amount of hops (N), the inactivity period of the mode (T_PARK_), the ratio of inactivity period affecting the latency (α), and the minimum hop latency (δ_min_), which summarizes not only the time for processing the data-packets but also other minor contributions. Furthermore, the probability of a retransmission in the multi-hop communication happening is also included in Equation (1): when one retransmission happens (*p_1_(retx)*), two (*p_2_(retx)*), and more. It happens not only with ping data-packets but also with pingback data-packets. 

(1) $$\delta_{PARK}=N\cdot (\alpha \cdot T_{PARK}+\delta_{min})+p_{1}(retx)\cdot \alpha \cdot T_{PARK}+p_{2}(2retx)\cdot 2\cdot \alpha \cdot T_{PARK}+...$$

After that, the values for the coefficients in Equation (1) were calculated through linear multiple regression. Since the probability values (*p_1_(retx), p_2_(2retx)*, etc.) depend on the actual deployment of the home automation application, they have not been included in the regression. Then, the curves that best fit to the results of the tests summarized in Table 1 for the park mode were defined. 

The performance in terms of latency of the multi-hop communication network for the ping data-packets when links are in the park mode can be modeled with Equation (2). When the communication is from slave to master, i.e., with pingback data-packets, the performance is similarly modeled with Equation (3). 

(2) $$\delta_{PARK}(ping)=N\cdot (0.34\cdot T_{PARK}+0.249)+0.191$$

(3) $$\delta_{PARK}(pingback)=N\cdot (0.33\cdot T_{PARK}+0.265)+0.283$$

After obtaining the values for the coefficients in Equation (1), the models for the multi-hop communication when working with links in the park mode were validated with R^2^ statistical datum. The values of R^2^ factor for the results summarized in Table 1 and the models defined by Equations (2) and (3) are 0.9527 when considering ping data-packets, and 0.9396 when considering pingback data-packets. Moreover, the MSE for Equation (2) is 0.091 and for Equation (3) is 0.089. 

As a summary, the latency in asynchronous links of Bluetooth multi-hop networks can be defined with just one equation for each one of the directions, i.e., for ping and pingback data-packets, in park low power mode. Furthermore, the two models were validated with the R^2^ statistical parameter and the MSE. 

### 6.2. Bluetooth Sniff Low Power Mode

Taking Table 2 as reference, the latency from end-to-end in Bluetooth multi-hop networks when links are always in sniff low power mode can be modeled as in Equation (4), where some of the time contributions described in [40] are included, namely the amount of hops (N), the inactivity period of the mode (T_SNIFF_), the ratio of inactivity period affecting the latency (β), and the minimum hop latency (δ_min_), which summarizes not only the time for processing the data-packets but also other minor contributions. Furthermore, the probability to happen a retransmission in the multi-hop communication is also included in Equation (4): when one retransmission happens (*p_1_(retx)*), two (*p_2_(retx)*), and more. It happens not only with ping data-packets but also with pingback data-packets. 

(4) $$\delta_{SNIFF}=N\cdot (\beta \cdot T_{SNIFF}+\delta_{min})+p_{1}(retx)\cdot \beta \cdot T_{SNIFF}+p_{2}(2retx)\cdot 2\cdot \beta \cdot T_{SNIFF}+...$$

After that, the values for the coefficients in Equation (4) were calculated through linear multiple regression. Since the probability values (*p_1_(retx), p_2_(2retx)*, etc.) depend on the actual deployment of the home automation application, they have not been included in the regression. Then, the curves that best fit to the results of the tests summarized in Table 2 for the sniff mode were defined. 

The performance in terms of latency of the multi-hop communication network for the ping data-packets when links are in the park mode can be modeled with Equation (5). When the communication is from slave to master, i.e., with pingback data-packets, the performance is similarly modeled with Equation (6). 

(5) $$\delta_{SNIFF}(ping)=N\cdot (0.45\cdot T_{SNIFF}+0.033)+0.202$$

(6) $$\delta_{SNIFF}(pingback)=N\cdot (0.46\cdot T_{SNIFF}+0.042)+0.189$$

After obtaining the values for the coefficients in Equation (4), the models for the multi-hop communication when working with links in the park mode were validated with R^2^ statistical datum. The values of R^2^ factor for the results summarized in Table 2 and the models defined by Equations (5) and (6) are 0.9927 when considering ping data-packets, and 0.9910 when considering pingback data-packets. Moreover, the MSE for Equation (5) is 0.023 and for Equation (6) is 0.057. 

As a summary, the latency in asynchronous links of Bluetooth multi-hop networks can be defined with just one equation for each one of the directions, i.e., for ping and pingback data-packets, in park low power mode. Furthermore, the two models were validated with the R^2^ statistical parameter and the MSE. 

### 6.3. The Most Suitable Low Power Mode for Any Application 

It is a fact that there is not only one low power mode that provides the lowest latency. The choice depends on the amount of hops included in the communication. The value of the inactivity period that matches the latency when working in park and sniff power modes can be calculated from the models defined in Section 6. The value of the inactivity period is extracted from the equation that defines that match. Two cases are considered: with ping data-packets (i.e., the data traffic goes from master to slave), and with pingback data-packets (i.e., the data go from slave to master).

**Figure 5:**
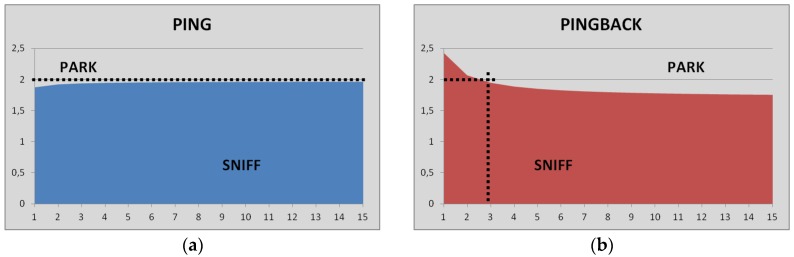
Curve where the latency in the sniff mode matches the one in the park mode for both directions of communication: (**a**) ping data-packets for master-to-slave communication; and (**b**) pingback data-packets for slave-to-master communication.

The curve where the latency in the sniff mode equals the one in the park mode when ping data-packets are used, i.e., for master-to-slave direction of communication is shown in Figure 5a. The vertical axis represents the inactivity interval of the low power mode (T) measured in s, and the horizontal axis the amount of hops involved in the end-to-end communication (N). Below the limit curve sniff gives smaller latency, but above the curve, it is the park mode the fastest one. In the same way, Figure 5b shows the same limit for the sniff mode when pingback data-packets are used. Furthermore, the horizontal dotted line represents the range of period of inactivity recommended by the chipset manufacturer, i.e., up to 2 s. 

In the range of period of inactivity recommended, i.e., up to 2 s, when the multi-hop communication takes place from master to slave (ping data-packets), the park mode is always the best option for the amounts of hops depicted in Figure 5a, as it provides lower values of latency than the sniff mode. On the contrary, if the multi-hop communication takes place from slave to master (pingback data-packets) and the inactivity interval is 2 s duration, the park mode is the best option in terms of latency, when three or more hops are included in the multi-hop communication. As a summary, for inactivity interval below 1.5 s duration, the sniff low power mode provides the best latency results in Bluetooth multi-hop networking. 

## 7. Conclusions

The vast literature about Bluetooth features a gap relative to the analysis of the latency in multi-hop communications. This QoS metric depends on several factors. Thus, this paper deals with the dependences of latency with the major factors, namely the hop count, the power mode of the nodes (sniff and park) and the period of inactivity. 

First, we implemented several Bluetooth nodes for the test bench in order to analyze the latency in the multi-hop wireless network. Furthermore, we interconnected the nodes and deployed the test bench to carry out the tests. Next, we applied a specific innovative methodology to measure the end-to-end latency along the data transportation network. In addition, the data mining of the results of the tests lead to the main findings for the end-to-end latencies with each one of the power modes, and their dependence with the hop count and period of inactivity of the mode. 

The main outcome of the paper is the set of equations obtained through linear regression to describe the low power modes in Bluetooth multi-hop wireless networks in terms of latency. To the best of our knowledge, this is the first approach to the end-to-end latency modeling in Bluetooth multi-hop communications when nodes are in sniff and park low power mode. The equations ease the calculation of the latency in communications over ACL links in scatternets when links are in park and sniff low power modes. They benefit the designers of not only home automation systems but also wireless networks. However, the set of equations do not consider further processing in the nodes of the network, so the additional latency due to extra processing tasks must be added to the latency given by the equation in the model. Moreover, it is important to take into account that the probability for retransmissions during the multi-hop communication could not be negligible, as shown in the tests. 

Finally, in future research, it is our intention to enhance the introduced model to be more accurate by extending the topology and the network application scenario considered as well as by using devices of the most recent release of Bluetooth standard. 

## Figures and Tables

**Table 1:** Latency vs. amount of hops (links always in the park mode). Inactivity interval (T_PARK_) ranges from 2 s to 0.1 s.

N	T_PARK_	δ_PING_	δ_PINGBACK_
0	2	0.20	0.30
1	2	-	1.10
2	2	1.50	-
3	2	2.65	2.70
4	2	3.70	3.70
5	2	4.50	4.70
6	2	5.40	-
0	1	0.20	0.30
1	1	-	1.10
2	1	1.50	1.95
3	1	2.30	2.70
4	1	3.00	3.30
5	1	3.60	4.20
6	1	4.30	-
7	1	4.90	-
0	0.5	0.20	-
1	0.5	-	0.60
2	0.5	-	0.90
3	0.5	-	1.20
4	0.5	1.90	1.60
5	0.5	2.40	2.20
6	0.5	2.90	2.50
7	0.5	3.40	-
0	0.1	0.20	0.20
1	0.1	0.40	0.50
2	0.1	-	0.80
3	0.1	-	1.20
4	0.1	-	1.50
5	0.1	1.50	-
6	0,1	1.70	-
7	0.1	1.90	-
8	0.1	2.10	-
9	0.1	2.40	-

**Table 2:** Latency vs. amount of hops (links always in the sniff mode). Inactivity interval (T_SNIFF_) ranges from 2 s to 0.1 s.

N	T_SNIFF_	δ_PING_	δ_PINGBACK_
2	2	2.10	2.00
3	2	3.00	3.10
4	2	4.30	4.30
5	2	5.10	5.10
6	2	5.80	5.80
7	2	6.30	6.30
2	1	1.20	0.90
3	1	1.60	1.50
5	1	2.60	2.50
6	1	3.05	3.10
7	1	3.60	3.50
8	1	4.10	4.05
1	0.5	-	0.40
2	0.5	0.70	0.60
3	0.5	1.05	-
4	0.5	-	1.00
5	0.5	1.50	1.20
6	0.5	1.90	-
7	0.5	-	1.60
8	0.5	2.40	
2	0.1	0.20	0.30
3	0.1	0.30	0.40
4	0.1	0.40	0.50
5	0.1	-	0.60
6	0.1	0.60	0.70

## Abbreviations

The following abbreviations are used in this manuscript:

QoS: Quality of Service
COTS: Commercially available Off-The-Shelf
HCI: Host Controller Interface
ACL: Asynchronous Connectionless Links
AN_i_: Access Node
MN_ij_: Mobile Node
DS: Data Sink
NW: Network
TDM: Time Division Multiplex
GFSK: Gaussian Frequency Shift Keying
BER: Bit Error Rate
FTD: File Transfer Delay
ANE: Addressed Node Emulator
MSE: mean squared error
